# Supplementary material for: Virus Pop—Expanding Viral Databases by Protein Sequence Simulation
Source: Viruses. 2023 May 24;15(6):1227. doi: 10.3390/v15061227 (PMC10304111; doi:10.3390/v15061227)
Supplement: Supplementary file 1 [file viruses-15-01227-s001.zip › S2_sarbecovirus_spike_structural_evaluation/sarbecovirus_spike_simulations_clustal_and_binding_energy.pdf]

```
                                                                      ↓
SARS-CoV-2       RVQPTESIVRFPNITNLCPFGEVFNATRFASVYAWNRKRISNCVADYSVLYNSA-SFSTFKCYGVSPTKLNDLCFTNVYADSFVIRGDEVRQIAPGQTGKIADYNYKLPDDFTGCVI
Node65_4_0.1     ...........................T........................NTL.....................S.......V..............R.................
Node209_1_0.2    ..K.SQ.VA.Y.........PQ.....K.P.....M.E...Y.I....I....TL.....R..............S......M.VK.....R...S...V............S...L
Node209_3_0.3    ..A..Q.VA.Y.....I...SQ.....N.P.....T.E.....I...TI...LTA...N.R..............SA.....M.VR.E.......S...V......R........IV
Node209_1_0.5    .SA.SLTVA.Y.........NQI...N..P.....T.Q.....L...NIF...QS.....R..............SR.....L.V.......KT......V.........N..N...L
Node209_2_1      ..V.GKQVA.T.....A...TS....VN.TP....T.EI..T.S...AII..TTV.....R.........D....SQ.....IAV.V...N....SE..V.VN....Q.........
Node66_3_1       .SK.M..TLK.....G..........IS..T....S.QK..S.....T.I..GTENL...R......S.......SK.....L.V.S.K.S.....K..H......E.....Q..I.
Node208_1_0.2    ..A.SVEV..I..........Q.....S.P.....E.D..SD.......F..GTP.Y..................SS.......V.E.........R..V................L
Node208_2_0.2    ..S..NEV..I....K...........S.P.....D....S............TE.....R.........H....S.....F..V..............I................L
Node208_2_0.5    ..N..FGVLSS.....V...D.....NQ.P.....E.T..YS...........TN.....R..............SD....A.AA..E..KR.......I..........A.....S

SARS-CoV-1       ..V.SGDV...........................K.P.....E..K...............T-F..........A........S........VK..D..........V............M...L
Node71_2_t0.1    .IS..KE..............P.....T..........R.............G.KS.............................................................
Node70_3_0.3     .QP.TSEM..................MD..........L........NI.....SS..........S........M........AR..E...M...................N...V
Node70_4_0.5     ..S.AKG.............SD....SS..T......IQ..D..........N.ES....N.....P........T.........R..E.I.T......L..........V.N...V
Node102_4_1      ....IWTAF.IL.V.TR....KI...AV..G....D.T..GT..T..A....R.VA....E.....PS..I....TY....T.LVRFSA.K.T...D............E..A....
Node219_4_1      .A..WTPI..T....S.....RI...NT.QI......MR....M...NIV..ASEV.H..R.....PS........S....YI..RIED.......D..M............S...P

Conserved aa     *  *        *:*  ***  :***  *  **** *  *  * :** :.**      .*.*****.:** ****  **** :  : . * : **. ** *.:***. *: * **:

                                                                ↓                   ↓
SARS-CoV-2       AWNSNNLDSKVGGNYNYLYRLFRKSNLKPFERDISTEIYQAGSTPCNGVEGFNCYFPLQSYGFQPTN-GVGYQPYRVVVLSFELLHAPATVCGPKKSTNLVKNKCVNF            -10,386
Node65_4_0.1     ....DE..T........S......A.........................K....L...T.A.H...Y...R.............N......................    12/17   1,226
Node209_1_0.2    ...IGAA.DSGSH.WQ.F.....D.K.L..Q....NVR.GF.KSNGCKPPSSD..WI.TK...ASGVLSQD...F..........N.........R..E..Y.R....     6/17   -10,775
Node209_3_0.3    ...ARSK.ENE.S.GK.F...Q...K.L..Q..V.NVA.GT.KRDYC.PPEAD..W..TN...SVSVRSQDF.............N.........R..V..YRA....     5/17   -9,267
Node209_1_0.5    ....QAK.ANG.H.WV.F..Q..N.R.S..Q..V.NVM.G.KKDDGCNPNHKD..W...K.R.KRSAFSQK...S..........N.........R..D..Y.N....     7/17   -7,154
Node209_2_1      ....RID.NHDT..DKFI...I.LGA.T.VI..LINYK.G..GNDGCVPKENG..GL.IR.S.RVDRDSHIS.P...........N.........S.....L.Q....     4/17   -3,406
Node66_3_1       ...NETV.VSWVE.RE.I...M.....RS....M.R.EFSQSGIFCQSI..WS..A.....K.RQL.M...F.SH..........G.........E..R....N....     6/17   14,649
Node208_1_0.2    ...TRK..ARQ....S.Y.....HGK.........NVW.SP.EK..S...E....Q..AN...APR.I.I...............N.........Q..I.........    12/17   -13,77
Node208_2_0.2    ...TKK..GRME...G.Y.....RGK.T.......NIT.STEGWS.SSSGN.I..WS.SL...TG.TK.IS...............N.........Q..E.........    10/17   -10,453
Node208_2_0.5    ...AST..EREY..LG.Y..IT..RK.........NIL.GV.DSH.DQTSRY.L.W..AG...TGIRG.I................D.........H..G...DR....     6/17   -5,956

SARS-CoV-1       ...TR.I.ATST.......K..YL.HGK.R.......NVPFSPDGK..T-PPAL...W..ND...YT.T-.I...............N.........L..D.I..Q....
Node71_2_t0.1    ......L....S..FD....SV.Q.........M.........N...V...F...........E.N.I........................................     9/14   -11,669
Node70_3_0.3     ....KH..TSKR..FT.Y..S..YS.IG.K......I.YL.G.QQ.VTQKKQ......SS.V.FP.SV.........................Y..N.V.S.....     7/14   -4,701
Node70_4_0.5     ......V.NGK....Y.Q..S..T.....Y....T.MGSNVGDF..RKTE.V....S.F.....SIMF.Y.F..N...........T.....................     8/14   -12,823
Node102_4_1      ....NPV.SDYRP.GS.N..NM.GA..K.YA..Q.S.RS.QG.DS.GTRNPT.GVRT..Q.A.LVLVYPITF.AA..........T............Q.........     4/14   10,986
Node219_4_1      ...SHY..SYHIS.TRFL..MF.F...D.MN..T...LYY.IESE.FTVLGNK..ML.KG...VSPPH.GRW..C.......G...T.........R..K.V..K....     4/14   -8,862

Conserved aa     ***    *     *  : **  *   : .  **                        *  * *         *. ******* ** :******** ** *:   ****    Conserved contact residues   Binding energy [kcal/mol]
```
