# Supplementary figures and images for: Virus Pop—Expanding Viral Databases by Protein Sequence Simulation
Source: Viruses. 2023 May 24;15(6):1227. doi: 10.3390/v15061227 (PMC10304111; doi:10.3390/v15061227)

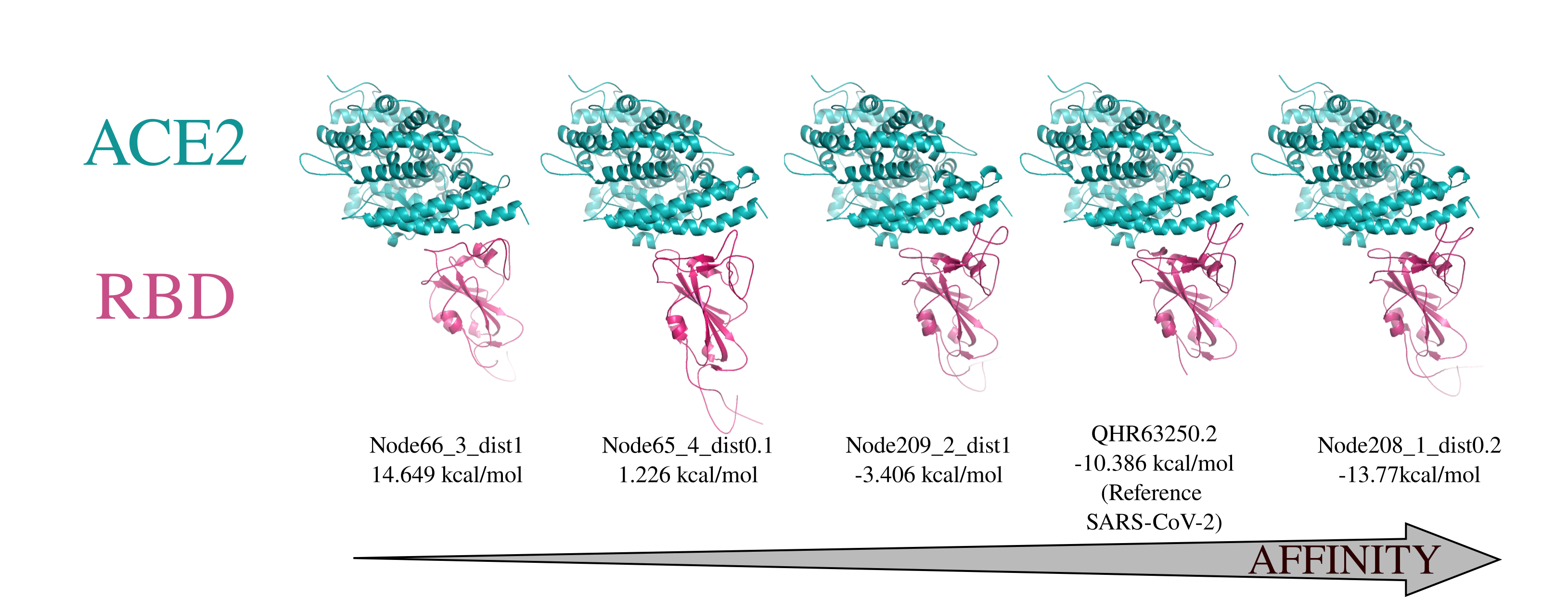

Supplement: Supplementary file 1 [file viruses-15-01227-s001.zip › S2_sarbecovirus_spike_structural_evaluation/structure.png]
